# Intestinal microbiota composition and bile salt hydrolase activity in fast and slow growing broiler chickens: implications for growth performance and production efficiency

**DOI:** 10.1186/s40104-025-01243-4

**Published:** 2025-08-02

**Authors:** Hye Won Kim, Na Kyung Kim, Patricia G. Wolf, Kristoffer Brandvold, Joshua M. Rehberger, Tom G. Rehberger, Ryan N. Dilger, Alexandra H. Smith, Roderick I. Mackie

**Affiliations:** 1 Department of Animal Sciences, University of Illinois Urbana-Champaign, Urbana, IL 61801 USA; 2 College of Health and Human Sciences, Purdue University, West Lafayette, IN 47907 USA; 3 Department of Nutrition and Exercise Physiology, Elson S. Floyd College of Medicine, Washington State University, Spokane, WA 99202 USA; 4 Arm & Hammer Animal and Food Production, Waukesha, WI 53186 USA; 5 Carle R. Woese Institute for Genomic Biology, University of Illinois Urbana-Champaign, Urbana, IL 61801 USA

**Keywords:** Bile salt hydrolase activity, Broiler chickens, Fast growing, Gut microbiota, Slow growing

## Abstract

**Background:**

Body weight is an important indicator of the overall health and production efficiency in broiler chickens. In broiler houses, body weight of chicks is variable despite the same genetics, hatching and feeding practices within a production system. The objective of this study was to investigate the intestinal microbiota and bile salt hydrolase (BSH) activity in slow and fast growing broiler chickens, which belonged to the 10^th^ and 90^th^ percentile body weight groups, respectively.

**Methods:**

A total of 300 Ross 308 broiler chickens (100 per cohort from three independent cohorts) were selected and mucosal samples from the jejunum, ileum, and cecum were collected at day of arrival, 11 and 25 (*n* = 450). Then, bacterial counts, 16S rRNA amplicon sequencing, species specific real-time qPCR, as well as BSH activity were analyzed.

**Results:**

Results of bacterial counts showed no significant difference between slow and fast growing cohorts (*P* > 0.05), but they tended to be higher in the slow growing chickens in all measured bacterial groups in cecum. The 16S rRNA amplicon sequencing revealed higher relative abundance of *E. coli-Shigella* (71.3%−79.8%) at day of arrival, while the most abundant microorganisms at d 25 was Candidatus *Arthromitus* (slow: 44.5%; fast: 27.4%) in small intestine. qPCR results indicated significant differences in bacterial populations between the slow and fast growing chickens, especially higher total bacteria, *Enterococcus*, and *Clostridium* cluster I in the slow growing chickens at d 25. BSH activity was higher in the slow growing chickens than the fast growing chickens [slow: 0.476 ΔOD/protein (μg/mL); fast: 0.258 ΔOD/protein (μg/mL); *P* < 0.0001], and correlation analysis highlighted associations between BSH activity, body weight, feed intake, body weight gain, and bacterial counts.

**Conclusions:**

We postulate that high total bacteria and *Enterococcus* abundance are associated with high BSH activity, impacting low feed intake and body weight gain, ultimately resulting in separation into slow and fast growing birds. The findings of this study contribute to understanding the relationship between gut microbiota, BSH activity, and host physiology in broiler chickens, with potential implications for poultry production.

**Supplementary Information:**

The online version contains supplementary material available at 10.1186/s40104-025-01243-4.

## Background

Chickens are one of the most widely farmed animals in the world, with a history of domestication over 5,000 years [1]. Since their early domestication, selective breeding and modern agricultural practices have altered chicken physiology, especially in broilers bred for rapid growth and high meat production [2, 3]. Today, modern commercial broiler chickens are generally raised to market weight within five to six weeks, as compared to the earlier chicken breeds used for broiler production that took much longer to reach that weight. In modern commercial production, broiler chickens are hatched in a clean hatchery environment in the absence of adult hens, raised indoors [4, 5] and they are managed to maximize their production by controlling diets, supplements, and light intensities to promote rapid growth [6, 7]. This rapid growth and maximized production have enabled the poultry industry to meet the increasing global demand for chicken meat [8, 9].

Body weight is one of the important indicators for assessing the production efficiency of the broiler chickens, as it directly reflects growth rate, health status, and overall meat yield [2, 10]. In the context of commercial poultry production, achieving target body weights within a short time is important for the production efficiency [11, 12]. In addition, maintaining a consistent body weight within groups of birds is important for processing efficiency, as well as for meeting market specifications. In broiler houses, however, despite the efforts to standardize the growing environment, there is still considerable variation in body weights of chickens within the same production system. This inconsistency of body weight among broilers with the same genetics and feeding practices initiated our investigations into the potential influencing factors, including the role of gut microbiota and the activity of bile salt hydrolase (BSH), an enzyme involved in bile salt metabolism.

Investigating chicken and gut microbiota interaction is of specific importance in terms of overall health and physiology of chickens. Chickens raised in commercial hatcheries are initially independent from adult hen microbiota [4] and gradually acquire their gut microbes from the surrounding environment where they are housed and grow [2]. This microbial succession is important, because variations in microbiota composition can lead to significant differences in health and productivity [2, 13]. Researchers have observed that differences in gut microbiota may correlate with body weight variations in host [2], suggesting that microbiota composition could be a key factor in understanding production variability of chickens.

BSH is an enzyme produced by specific gut bacteria, such as *Lactobacillus*, *Clostridium*, and *Enterococcus*, that can influence bile salt metabolism, with effects on fat digestion and nutrient absorption [14]. Specifically, the BSH enzyme catalyzes the deconjugation of conjugated bile acids by hydrolyzing the amide bond resulting in the production of free amino acids and unconjugated bile acid [15]. This activity decreases bile salt reabsorption in the intestine, which can impact cholesterol metabolism and energy balance in the host [14]. The impact of intestinal bacterial BSH activity on growth and health in food animals has been explored focusing on the animal production and health [16]. Variations in BSH activity, particularly higher BSH activity, have been associated with reduced growth rates due to diminished fat digestion and nutrient absorption efficiency. The abundance of potent BSH-producing bacteria, such as *Lactobacillus* and *Enterococcus*, plays a critical role in this process [17]. Understanding the relationship between gut microbiota, BSH activity, and broiler chicken physiology, therefore, could provide valuable insights for production practices.

The objective of this study was to investigate the intestinal microbiota and BSH activity in slow and fast growing broiler chickens in order to understand the potential links between gut microbiota, BSH activity, and host physiology in broiler chickens. To address this, we enumerated potential bacterial pathogens, performed microbiome analysis by 16S rRNA amplicon sequencing and real-time quantitative PCR (qPCR), and quantified BSH activity and then determined correlations between bacterial populations and BSH activity.

## Materials and methods

### Animal handling

All animal care and experimental procedures were approved by the University of Illinois Institutional Animal Care and Use Committee (IACUC protocol number 19093) prior to initiation of the experiment. Specific procedures of animal experiments are described in the previous study [18]. Briefly, Ross 308 broiler chickens, produced from a commercial hatchery (Hoover’s Hatchery, Rudd, IA, USA), were transported to the University of Illinois Edward R. Madigan Laboratory animal care facility. All chicks were raised in wire-bottom battery cages with continuous lighting and in-cage radiant heaters to regulate the microenvironment according to age-appropriate temperatures as recommended (Ross Broiler Management Handbook).

### Slow and fast growing chicken selection and sample collection

A total of 1,200 male Ross 308 broiler chickens were initially used, distributed equally across three independent cohorts (400 birds per cohort). To ensure uniformity, 100 chickens in each cohort showing the most consistent weight distribution were selected for further study. The average enrollment weights were as follows: cohort 1, 32 ± 2 g; cohort 2, 37 ± 2 g; cohort 3, 32 ± 2 g. To establish a baseline for physiological and microbial parameters, 10 chickens in each cohort study were randomly selected and euthanized at the day of arrival for comprehensive measurements of body weight, organ weights and length. All remaining chickens were housed in controlled temperature conditions and fed with a standard diet of corn and soybean meal (Table S1) for the entire 25-d study duration. At d 11 (early post-hatch period), all birds underwent weighing and then ranked based on individual weight gain percentiles from day of arrival to d 11. The 10^th^ percentile (slow-growing) and 90^th^ percentile (fast-growing) birds were identified per cohort. From each growth group in each cohort, 20 chickens (10 slow and 10 fast growers) were selected, totaling 120 birds (3 cohorts × 20 × 2 groups). Of these, 10 birds per group per cohort (5 slow and 5 fast growers) were euthanized at d 11 for mucosal sampling. The remaining 60 birds (10 per group per cohort) were placed into individual cages from d 11 to d 25 (mid-to-late growth phase) to monitor feed intake and body weight gain individually. These birds were euthanized at d 25 for microbiological and physiological analyses. In total, 150 chickens were sacrificed and 450 mucosal samples were taken from the gastrointestinal tract at three sites including the jejunum (from the end of the pancreatic ducts to Meckel’s diverticulum), the ileum (from Meckel’s diverticulum to the ileocecal junction), and paired ceca, which are key sites for nutrient absorption and microbial fermentation. Mucosal-associated microbiota are in closer contact with the host epithelium and play a direct role in modulating host metabolism. Therefore, for microbial analysis, mucosal samples were collected using sterile tools immediately after dissection to maintain aseptic conditions. The samples were then stored in a liquid nitrogen tank to preserve their integrity.

### Growth performance

Individual body weight, body weight gain, feed intake, and feed efficiency (measured as gain-to-feed ratio and feed conversion ratio) were assessed during the grower phase from d 11 to 25. At the end of this period, all birds were euthanized to allow for the collection of tissue and luminal content samples. Growth performance data are presented for both the slow and fast growing chickens.

### Bacterial counts

Mucosal scrapings of all samples were diluted 1:10 weight/volume in peptone. Serial dilution series were created and plated. Enumeration was calculated on a CFU/g of sample basis. *Escherichia coli* was enumerated on CHROMagar ECC (DRG International, New York, NY, USA). After being incubated for 24 h aerobically, five colonies were selected from each sample, DNA was extracted, and a multiplex PCR was performed containing primers for the virulence genes: *iroN*, *ompT*, *hlyF*, *iss*, and *iutA* [19]. If an isolate was found to be positive for two or more virulence genes that isolate was considered to be an avian pathogenic *E*. *coli* (APEC). The total *E*. *coli* counts were multiplied by the proportion of isolates found to be APEC from each sample to determine the APEC counts. *Clostridium* was enumerated on OXOID Perfringens TSC Agar Base (ThermoFisher Scientific Inc., Waltham, MA, USA) supplemented with egg yolk and d-cycloserine. Five colonies were selected from each sample, DNA extracted, and PCR performed to verify the presence of the *plc* gene [20]. The total *Clostridium* counts were multiplied by the proportion of isolates found positive for the *plc* gene for each sample to determine the *Clostridium perfringens* counts. Lactic acid bacteria (LAB) were enumerated on OXOID MRS Agar (ThermoFisher Scientific, Waltham, MA, USA). Perfringens TSC agar was incubated for 24 h in anaerobic boxes with AnaeroPacks (Mitsubishi Gas Chemical America, New York, NY, USA), and MRS was incubated for 48 h in anaerobic boxes with AnaeroPacks. All samples were incubated at 37 °C.

### DNA extraction, library preparation, and sequencing

Remaining 1:10 diluted mucosal scrapings were centrifuged at 10,000 ×* g* for 10 min. Supernatant was discarded and cell pellets were stored at −20 °C until DNA extraction.

Genomic DNA from small intestine and cecum was extracted using the DNeasy PowerSoil HTP Kit (QIAGEN, Germantown, MD, USA) with bead (0.7 mm garnet beads) beating for 2 min in a Mini-Beadbeater-96 (BioSpec Products, Bartlesville, OK, USA). Genomic DNA was used for library preparation: PCR was performed for 35 cycles (Table S2) with 16S rRNA V4 primers for total bacteria [21] using the 48 Access Array IFC (Fluidigm, San Francisco, CA, USA). Primers, dimers, and artifacts below 150 base pairs were removed by size selection. The final size-selected amplicon pools were quantitated using Qubit (Life Technologies, Grand Island, NY, USA) and then further quantitated by qPCR on a Bio-Rad CFX Connect Real-Time System (Bio-Rad Laboratories, Inc., CA, USA), then pooled evenly. The pool was loaded onto 1 lane of a 2-lane HiSeq Rapid V2 flowcell at a concentration of 10 pmol/L for cluster formation on the cBOT and then sequenced on an Illumina HiSeq 2500 with version 2 Rapid SBS sequencing reagents. The libraries were sequenced from both ends of the molecules to a total read length of 250 nt from each end.

### Sequencing data processing

Sequence analysis was performed using QIIME2 version 2021.2 [22]. Paired end sequences were merged, denoised, dereplicated and filtered of chimeras using DADA2 [23]. Taxonomy was assigned by closed-reference clustering using Vsearch [24] clustering at 97% similarity to the EZBioCloud reference database [25].

Alpha rarefaction plots of observed features were generated with a maximum sequencing depth of 15,000 reads to ensure a sufficient depth to cover the diversity within the samples was displayed. Alpha diversity as determined by Shannon Entropy was compared between groups and was measured at a depth of 6,650 sequences because it was deep enough to cover the diversity within each sample while retaining as many samples as possible in the analysis. All alpha diversity measurements were measured at ASV level before clustering and taxonomic assignment. Beta diversity was examined by Bray-Curtis dissimilarity and Jaccard index.

### Phylogeny analysis

A phylogenetic tree was constructed on the basis of the V4 region of 16S rRNA gene of Candidatus *Arthromitus* using MEGA-X version 10.2.6. The sequences of genome aligned using ClustalW, and phylogenetic trees were established using maximum likelihood method with a supporting bootstrap value of 1,000. The resulting gene sequences were filtered based on the confidence of the sequence (above 0.99) and the sampling sites. Phylogenetic trees were then visualized and annotated by iTOL version 6.9. The dataset includes baseline, d11_slow, d25_slow, d11_fast, d25_fast, and number of samples including that sequence. Clades were differentiated by color to show reference genome, baseline, slow growing, fast growing, and multiple groups.

### Volatile fatty acid analysis

Fresh luminal contents from the ileum and ceca were collected at d 25 to analyze concentrations of volatile fatty acids (VFA). Fresh samples were weighed and preserved immediately upon collection by adding an equal volume of 2 mol/L HCl, then stored at −20 °C for long-term preservation. Absolute VFA concentrations (mmol/g dry matter) were measured using gas chromatography (Hewlett-Packard 5890 A series 99) equipped with a custom packed column containing 10% SP-1200/1% phosphoric acid on 80/100 Chrom-WAW. The gas chromatography settings were injector at 175 °C, flame ionization detector at 180 °C, and oven at 125 °C. Relative molar ratios were calculated by dividing the absolute concentration of each analyte (mmol/g) by the total concentration, then multiplying by 100.

### DNA extraction for qPCR

Mucosal samples were extracted by gently scraping the inner lining of the gastrointestinal tract (from jejunum, ileum, and cecum of chickens) using sterile tools immediately after dissection. This process ensured the collection of mucosal material while minimizing contamination. Samples were then placed in a plastic centrifuge tube before experiments. Samples were cut (20 mg) and placed in the 1.5-mL Eppendorf tubes, and then used to extract DNA by the DNeasy Blood & Tissue kit (Qiagen Inc., CA, USA) according to the manufacturer’s instructions. Total of 450 DNA samples were then used to conduct the qPCR analyses in this study.

### Quantitative PCR

#### Empirical testing of the primers

The target bacterial taxa for the qPCR were total bacteria, lactic acid bacteria, *Lactobacillus,* Bifidobacteria*,* Enterobacteriaceae, *Enterococcus, Clostridium* cluster I, and *Bacteroidales*. Primer sequences used are presented in supplementary Table S3. *Lactobacillus fermentum* ATCC 9338, *Bifidobacterium suis* ATCC 27533, *Escherichia coli* NEB 316, *Enterococcus faecalis* ATCC 19433, *Clostridium perfringens* (R880.30.1 and R865.31.5 from Arm and Hammer), and *Prevotella ruminicola* 2202 were used as reference standards. Standard curves were established to quantify the target microorganisms. Briefly, reference cells grown on the nutrient medium or gut microbiota medium (GMM) [26] at 37 °C for 1–2 d were used to extract DNA using the QIAamp DNA Mini Kit (Qiagen) according to the manufacturer’s instructions. DNA was then serially diluted four-fold to generate a standard curve by qPCR. The regression equations of all the standard curves were presented in Table S4 for which R^2^ ranged from 0.91 to 0.99.

#### Fluidigm real-time qPCR

Each 384-well contained 20 μL reaction mixture with 10 μL of iQTM SYBR Green Supermix, 300 nmol/L of forward and reverse primers, 1 μL of DNA template, and 8 μL of pure H_2_O. The cycle conditions used in the Fluidigm real-time qPCR (Fluidigm Corporation, CA, USA) were as follows: 95 °C for 5 min, 40 cycles of 95 °C for 15 s and 60 °C for 1 min. Data from this experiment were collected through QuantStudio™ Real-Time PCR Software (ver. 1.3, Thermo Fisher Scientific, MA, USA). The Ct values obtained from the qPCR were calculated to the log copy number per gram of samples based on the standard curve established.

### Bile salt hydrolase activity

Bile salt hydrolase activity was examined based on the known properties of peptide aminocoumarin conjugates as previously described [27]. The BSH substrate used in this study was a synthetic compound, specifically the cholic acid-conjugated amino coumarin probe (CA-AMCA), which was synthesized by our co-author, K. Brandvold, at Washington State University. Briefly, the synthetic BSH substrate is hydrolyzed by the BSH and the released free aminocoumarin (fluorescent) is measured and normalized by the amount of total protein. For this measurement, 0.25 g of ileal content of chickens were mixed with anaerobic PBS and then the supernatant (650 μL) was lysed with glass beads (PowerBead Tubes Glass 0.1 mm, Qiagen) for 10 min at room temperature. Total 50 μL of lysate (1 mg/mL) with bile salt hydrolase (40 μg/mL) or buffer were located in the black 96-well microtiter plates (Thermo Scientific). Then a 50 μL solution of probe (CA-AMCA, 300 μmol/L, synthesized in the Washington State University) in buffer with 5% DMSO was added to initiate the reaction. Reactions were processed for 24 h at 37 °C and monitored at 450 nm (excitation 350 nm). The concentration of BSH was determined using the BCA protein assay kit (Thermo Scientific) following the manufacturer’s instructions. Bovine serum albumin was used as the protein standard. *Clostridium scindens* ATCC 35704 and *Bacteroides vulgatus* ATCC 8482 were used as negative and positive controls, respectively. All experiments were performed in triplicate.

### Statistical analysis

Plots were generated by R-Studio ver. 1.4.1717, SigmaPlot ver. 12.5, and Excel 2016. Statistical analysis was performed using a SAS (version 9.4; SAS Institute; Cary, NC, USA). Growth performance data were analyzed using a one-way ANOVA with the MIXED procedure in SAS. qPCR data were evaluated by a general linear model for variance analysis and Tukey’s post Hoc test was used to determine the significance of the differences among samples (*P* < 0.05). Correlation analysis between BSH activity vs. body weight gain, feed intake, total bacterial counts, or *Enterococcus* counts were performed using Pearson correlation analysis.

## Results

**Fig. 1:**
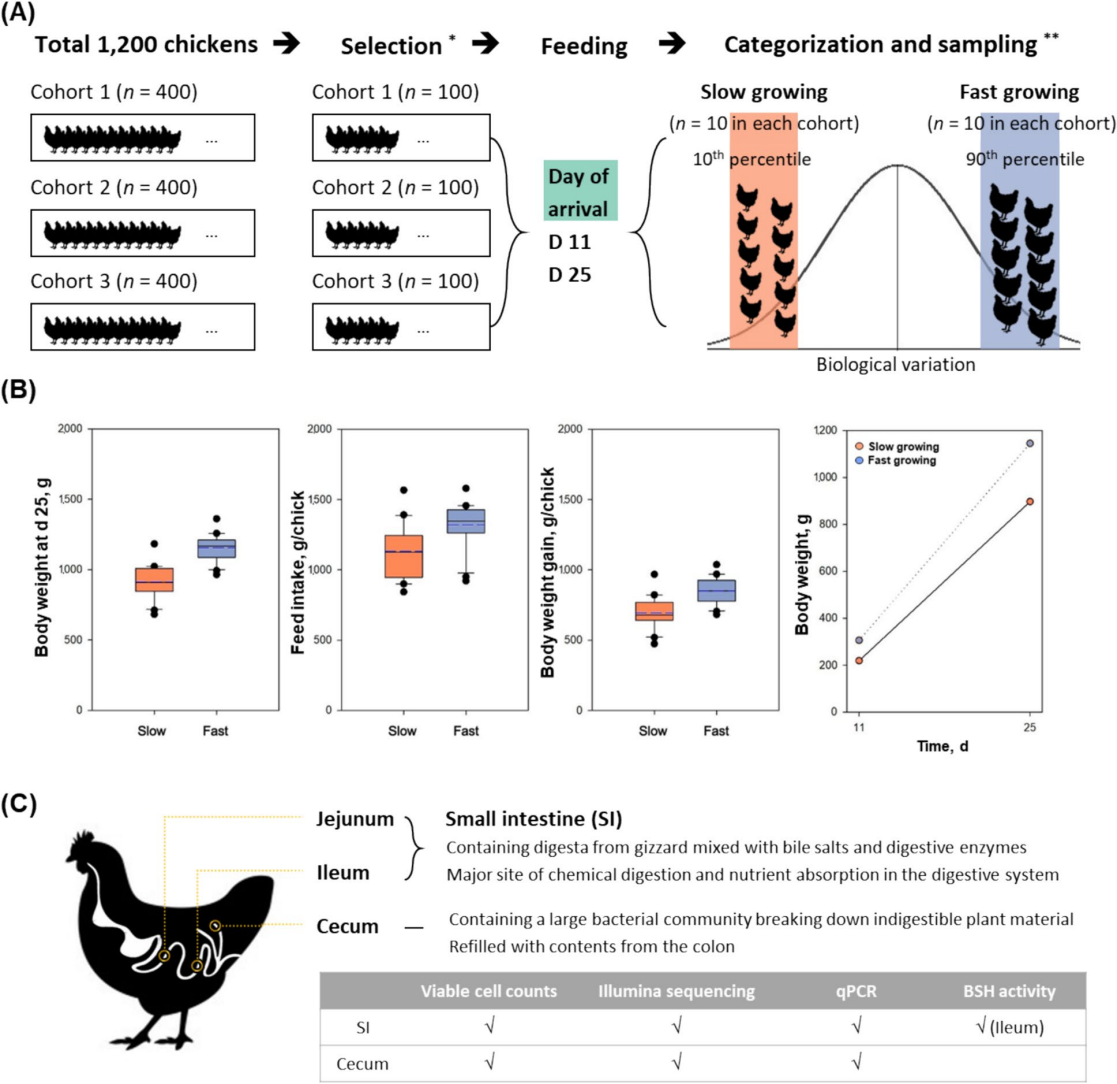
Schematic of chicken sampling, body weight, feed intake, and sampling sites. **A** Schematic diagram to illustrate chicken handling and sampling. ^*^ A total of 300 chickens showing the most consistent weight distribution and minimal body weight change were selected. A random selection of 10 at the day of hatch was utilized for the day of arrival (green). ^**^ Slow (red) and fast (blue) growing chickens were sampled at d 11 and d 25. Total chicken samples used in this study were 150. **B** Body weight at d 25, feed intake (g/chick) between d 11 and d 25, body weight gain (g/chick) between d 11 and d 25, and body weight changes between d 11 and d 25 in cohorts of slow and fast growing chickens. **C** Three sampling points of gastrointestinal tract (jejunum, ileum, and cecum). A total 450 mucosal samples were taken and analyzed

Ross 308 chickens are widely recognized for their fast growth rate and overall stable performance under controlled commercial conditions. However, within a given flock, individual variations in growth can still occur due to factors such as microbiota composition, metabolism, and feed efficiency, which this study aimed to investigate. In this study, we received 400 chickens 2 d after hatch from a commercial hatchery for three different times (total 1,200 chickens, Fig. 1A). After measuring the body weight of all chickens, we selected 100 chickens in each cohort. We randomly selected 10 chickens in each cohort to use as a baseline, the starting point of this research (day of arrival, baseline highlighted in green in Fig. 1A). Growth performance including body weight, feed intake, body weight gain, gain:feed, and feed conversion ratio is presented in Fig. 1 and Table S5. At d 11, we collected 20 chickens in each slow (red) and fast (blue) growing cohorts of which body weight gain percentiles were closest to the 10^th^ and 90^th^ (average body weight: slow, 219 g; fast, 306 g). Half of them were sacrificed for the analysis and the other half were individually caged and then sampled at d 25 (slow, 897 g; fast, 1,145 g; *P* < 0.001) (Fig. 1B). Therefore, a total of 150 chicken samples were used in this study, including 30 baseline samples, 20 samples each from the slow and fast growing chickens at two time points and three cohort studies.

While all chicks received the same antibiotic-free diet under the same environmental conditions, nevertheless, body weights of chickens were variable and fast growing chickens showed significantly higher body weight. When comparing body weight gain, fast growing chickens showed a faster growth rate than the slow growing chickens (Fig. 1B), which indicates slow growing chickens were unlikely to catch up to the fast growing chickens. To understand the possible impact of gut microbiota on these body weight differences, we analyzed mucosal sample of small intestine (jejunum and ileum) and cecum (Fig. 1C). Therefore, in total, 450 mucosal samples were collected from the chicken specimens and subsequently analyzed.

**Fig. 2:**
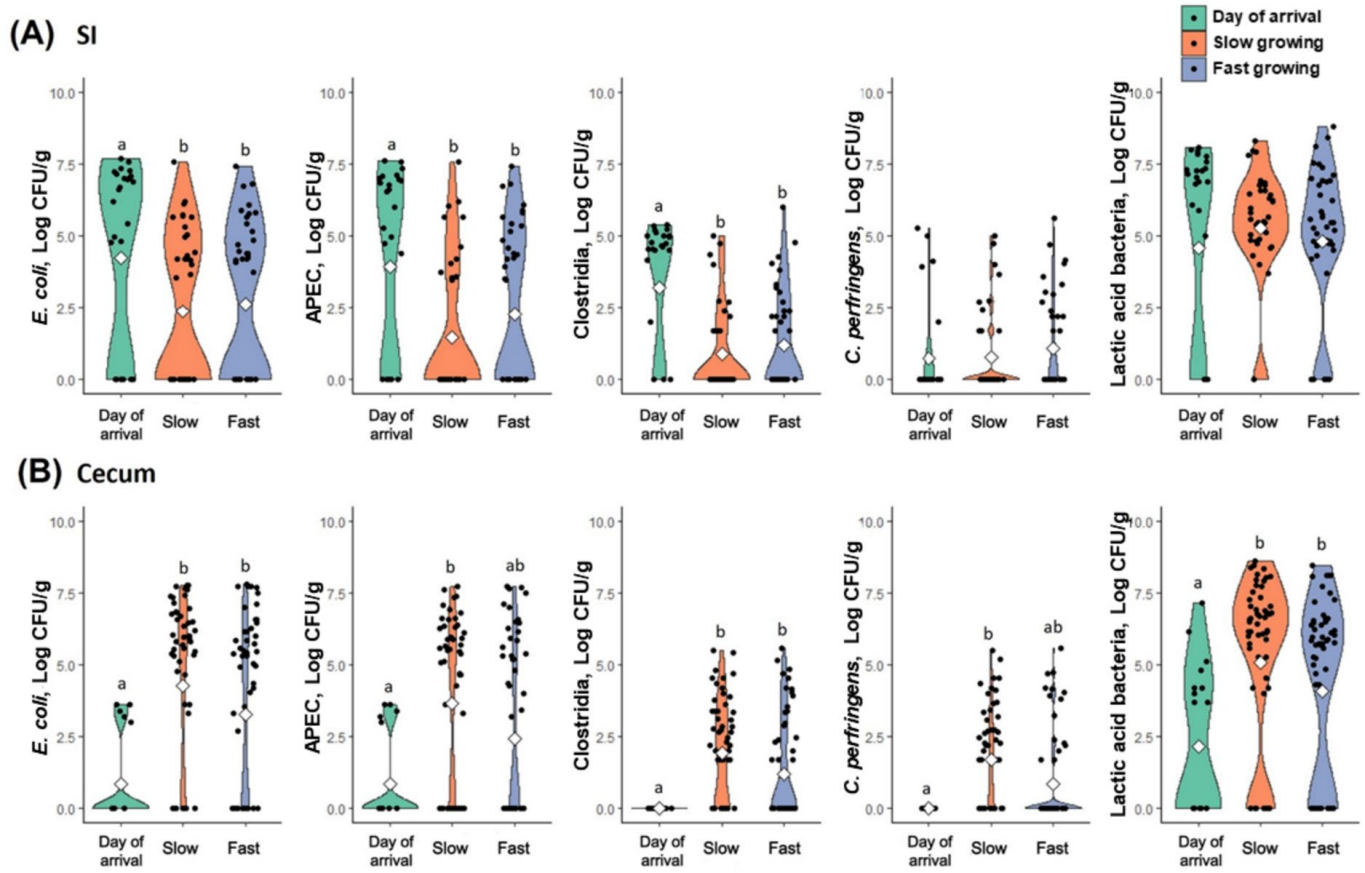
Viable cell counts (log CFU/g) of intestinal bacteria in chicken small intestine and cecum. *E. coli*, Avian pathogenic *Escherichia coli* (APEC), *Clostridia*, *C. perfringens*, and lactic acid bacteria in (**A**) small intestine (SI) and (**B**) cecum in each group (day of arrival, slow growing, and fast growing) were measured by plate counting methods and identified by PCR. The white diamond within the violin plots represents the mean values. ^a,^^b^Different letters above the violin plots indicate significant differences (*P* < 0.05)

Figure 2 shows viable cell counts of *E. coli*, APEC, *Clostridia, C. perfringens*, and lactic acid bacteria in the small intestine and cecum for each group (day of arrival, slow, and fast growing). In small intestine, *E. coli*, APEC, and *Clostridia* were significantly higher in the baseline (*P* < 0.05), indicating decrease by time. However, there was no significant difference between the slow and fast growing cohorts at either d 11 or 25. In contrast, in the cecum, all measured bacteria were significantly higher in the slow and fast growing cohorts compared to the day of arrival (*P* < 0.05), indicating an increase in bacterial counts over time. While there was no significant difference between the slow and fast growing cohorts, bacterial counts tended to be higher in the slow growing chickens.

**Fig. 3:**
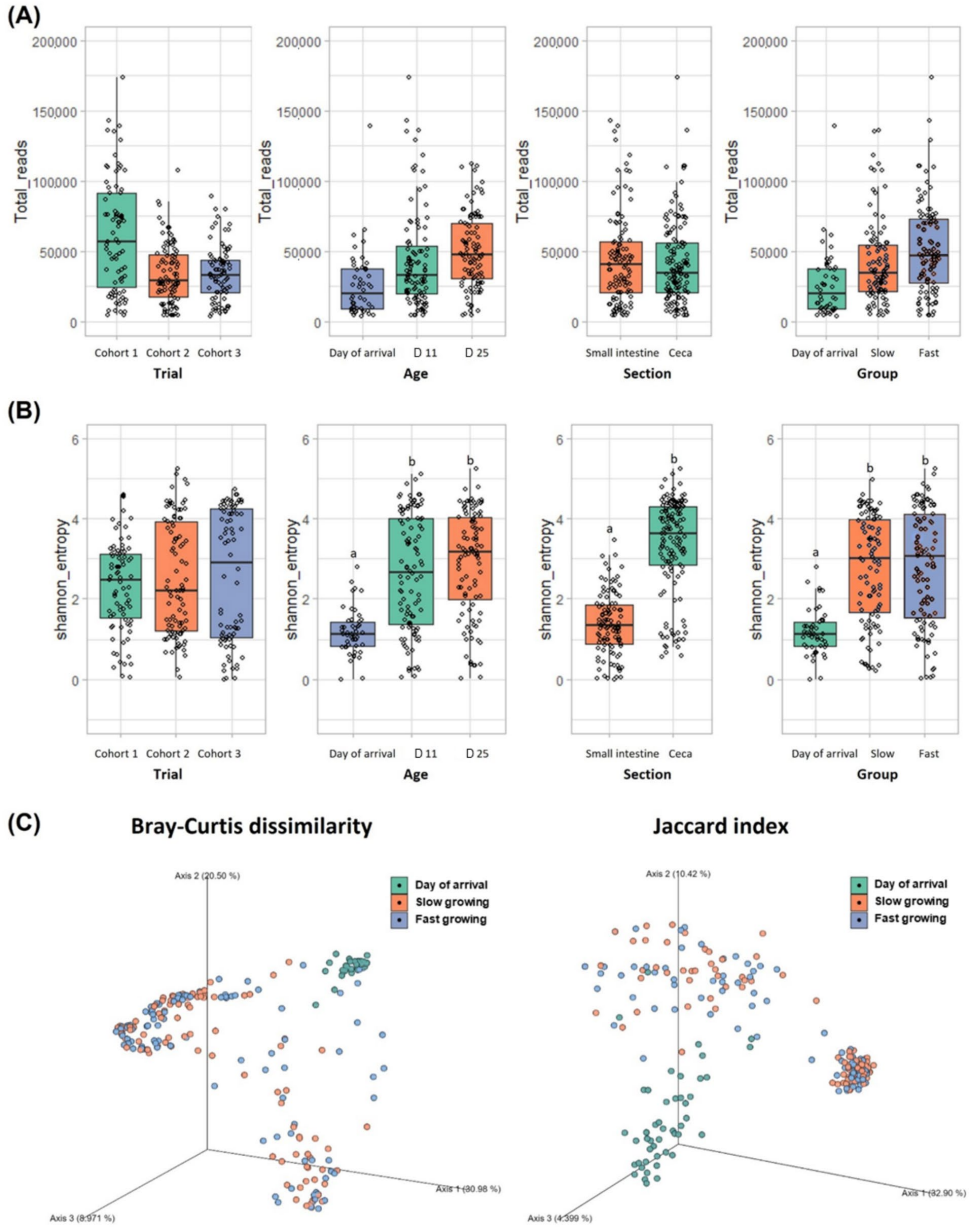
Microbial alpha and beta diversity in chicken intestinal samples. **A** Total reads and (**B**) alpha diversity indicated by the Shannon entropy in microbiomes of chicken samples of each trial (cohort study 1 to 3), age (day of arrival, d 11, and d 25), section (small intestine and ceca), and group (day of arrival, slow growing, and fast growing). ^a,b^Different letters above the box plots indicate significant differences (*P* < 0.05). **C** Bray-Curtis dissimilarity and Jaccard index showing beta diversity analysis among groups

To understand bacterial diversity in the intestinal samples of chickens, α- (Shannon entropy) and β-diversity (Bray-Curtis dissimilarity and Jaccard index) were calculated based on the 16S rRNA amplicon sequencing (Fig. 3 and Fig. S1). Shannon entropy was significantly higher at d 11 and 25 compared to day of arrival, higher in the ceca than in the SI, and higher in both the slow and fast growing cohorts compared to day of arrival (*P* < 0.05). However, there were no significant differences in alpha diversity between slow and fast growing cohorts as determined by Kruskal–Wallis test (*P* > 0.05). This indicates that age of chickens and location of intestinal tract are the most important factors affecting the bacterial diversity. In the PCoA plot calculated using the Bray-Curtis dissimilarity and Jaccard index, there was just a grouping differentiating baseline and slow/fast growing cohorts (*P* = 0.001 for both index), and there were no clear differences between the groups (*P* = 0.347 and 0.704 for Bray-Curtis and Jaccard index, respectively); although, taxonomic assignment showed some differences.

**Fig. 4:**
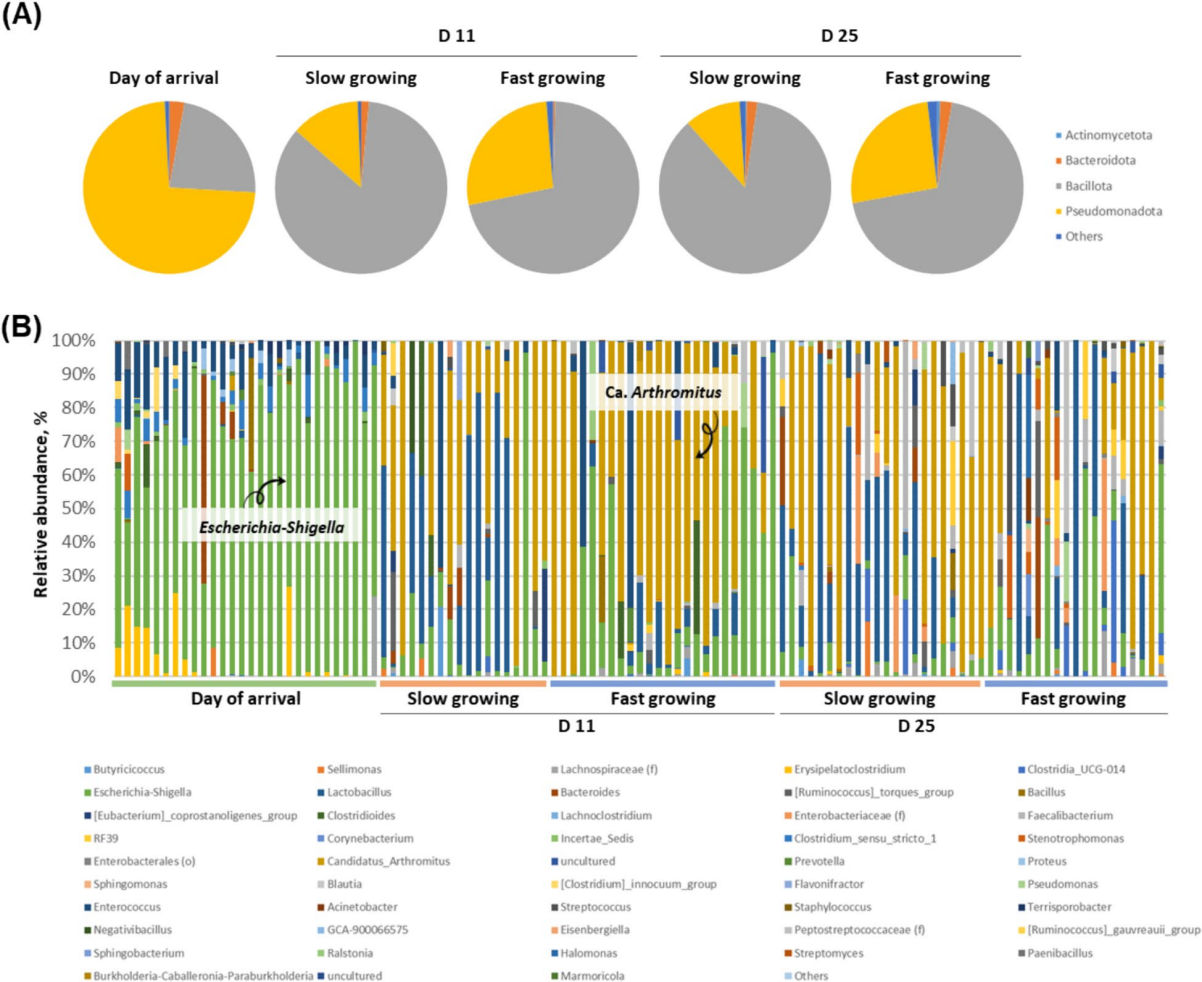
Relative abundance (%) of major bacteria in chicken small intestine microbiota. Data among different groups (day of arrival, slow growing, and fast growing) are shown at (**A**) phylum and (**B**) genus level

Figure 4A shows the relative abundance of microorganisms in small intestine at phylum level. At day of arrival, the most abundant bacteria were Pseudomonadota (formerly known as Proteobacteria). This pattern changed to Bacillota (former Firmicutes) which was higher in the slow growing chickens (84.96% and 86.10% at d 11 and 25, respectively) than in the fast growing chickens (71.35% and 69.35%, respectively; *P* < 0.05). At genus level (Fig. 4B), the abundant Pseudomonadota was identified as *Escherichia-Shigella* (71.3%) in day of arrival samples. During the growth phase, the most abundant taxa was Ca. *Arthromitus*, which tended to be higher in the slow growing chickens at the end of the experiment (at d 25, 44.5% and 27.4% in slow and fast growing chickens, respectively; *P* > 0.05). In fast growing chickens, Ca. *Arthromitus* was significantly more abundant at d 11 than at d 25 (52.8% and 27.4%; *P* < 0.05).

**Fig. 5:**
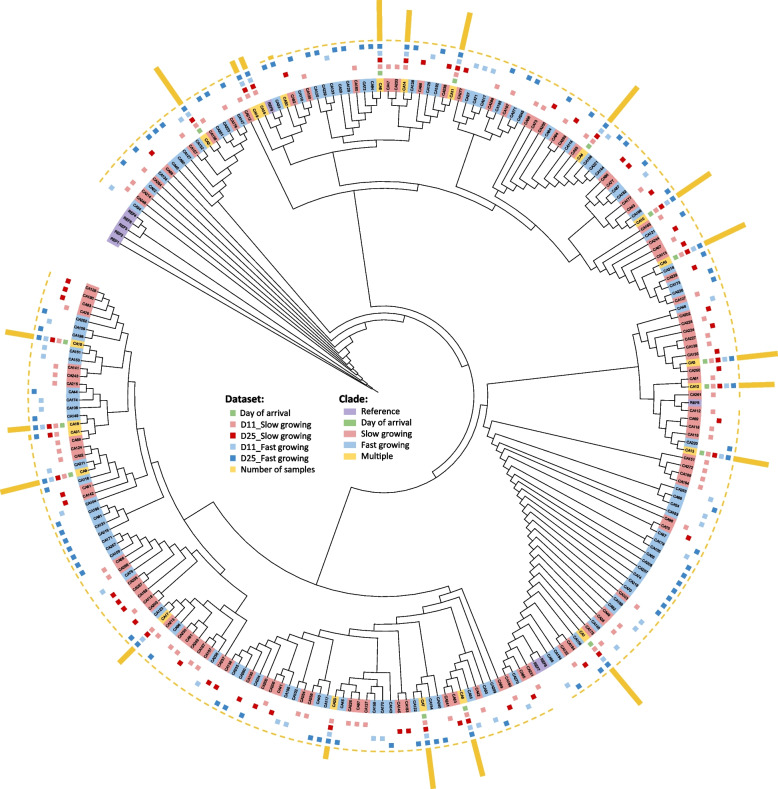
Phylogenetic tree of 16S rRNA assigned to Candidatus *Arthromitus*. Dataset includes day of arrival (green), slow (red) and fast (blue) growing chickens at each time points. Yellow bar indicates the number of samples including the 16S rRNA (REF1: X76748.1 *Clostridium colinum* 16S rRNA gene type strain DSM 6011T; REF2: L07416.1 *Clostridium piliforme* 16S rRNA gene sequence; REF3: KX659137.1 Ca. *Arthromitus* sp. SFB-mouse strain R5I-1380-9 16S rRNA gene partial sequence; REF4: NR 029230.1 *Clostridium beijerinckii* strain McCoyA 67 16S rRNA partial sequence; REF5: NR 042144.1 *Clostridium butyricum* strain VPI3266 16S rRNA partial sequence; REF6: KC135882.1 Ca. *Arthromitus* sp. SFB-human 16S rRNA gene partial sequence; REF7: NZ LXFF01000001.1 Ca. *Arthromitus* sp. SFB-turkey isolate UMNCA01 16S rRNA consensus whole genome shotgun sequence; REF8: D86305.1 Ca. *Arthromitus* sp. SFB-mouse gene for 16S rRNA partial sequence; REF9: D86302.1 Ca. *Arthromitus* sp. SFB-mouse gene for 16S rRNA partial sequence isolated from Fisher 344 rat)

In this study, we attempted to zoom into the 16S rRNA sequence of the taxa assigned to the Ca. *Arthromitus* population (Fig. 5). Based on the phylogenetic analysis, we found a broad range of dissimilarity suggesting that there is a diversity Ca. *Arthromitus* species and strains in the chicken gut. Compared to the 16S rRNA gene from different hosts including human, mouse, and turkey, there was no clear host specificity and no clear pattern by groups. Instead, some Ca. *Arthromitus* were found across all time periods from day of arrival to d 11 and d 25 (clade yellow) indicating the possible early colonization of this bacteria in the chicken gut at the hatchery. Since the previous study reported that Ca. *Arthromitus* is a BSH-carrying microorganisms, we assume that this microorganism is also related to the host physiology.

**Fig. 6:**
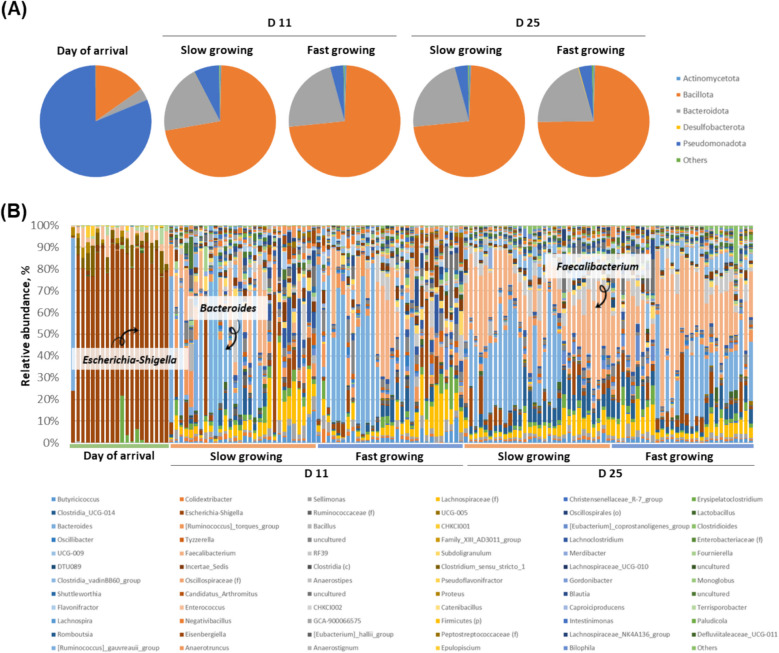
Relative abundance (%) of major microorganisms in chicken ceca samples. Data among different groups (day of arrival, slow, and fast growing) are shown at (**A**) phylum and (**B**) genus level

The relative abundance patterns of cecal bacteria were similar to that of the small intestine in that the most abundant taxa at phylum level was Pseudomonadota in day of arrival and Bacillota at d 11 and 25 in both slow and fast growing cohorts (Fig. 6A). The main difference to the small intestine microbiota was appearance of Bacteroidota as the second most abundant taxa in cecum at d 11 and 25. There was no clear differences between slow and fast growing chickens in cecum (*P* > 0.05). At genus level (Fig. 6B), *Escherichia-Shigella* was predominant again in day of arrival (79.8%). At d 11, *Bacteroides* (20.0%−22.3%) was abundant followed by *Faecalibacterium* (8.9%−10.1%), which was then reversed at d 25 (*Faecalibacterium*: 23.7%–25.7%). Especially, *Bacteroides* showed higher abundance in the slow growing chickens (19%) than in the fast growing chickens (12.0%) in cecum at d 25, while there was no significant difference (*P* > 0.05). In this study, we found higher acetate (Slow: 217.85 μmol/g DM, fast: 196.17 μmol/g DM in ileum, *P* < 0.001) and propionate (Slow: 17.54 μmol/g DM, fast: 1.41 μmol/g DM in cecum, *P* < 0.05) in the slow growing chickens (Fig. S2). This indicates that high abundance of *Bacteroides* in slow growing chickens could influence amounts of volatile fatty acid production.

**Fig. 7:**
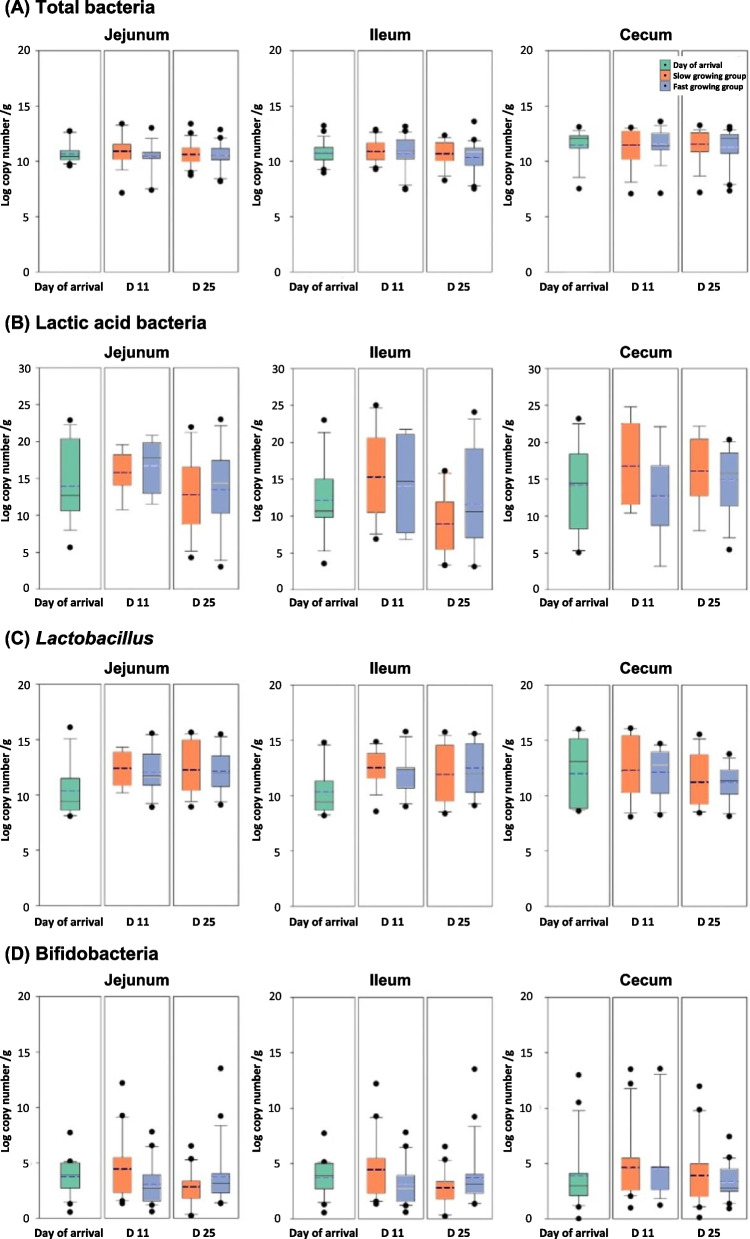
Bacterial populations in mucosa of the intestinal tract (jejunum, ileum, and cecum) analyzed by real-time qPCR. **A** Total bacteria, (**B**) lactic acid bacteria, (**C**) *Lactobacillus*, and (**D**) Bifidobacteria at day of arrival, d 11, and d 25. In box plots, square indicates the interquartile range for each data point, and the black and blue lines denote the median and mean values, respectively. The error bars represent the 10^th^ and 90^th^ percentiles and the black circles are outliers. ^a^^,b^ Different letters above the box plots indicate significant differences (*P* < 0.05)

**Fig. 8:**
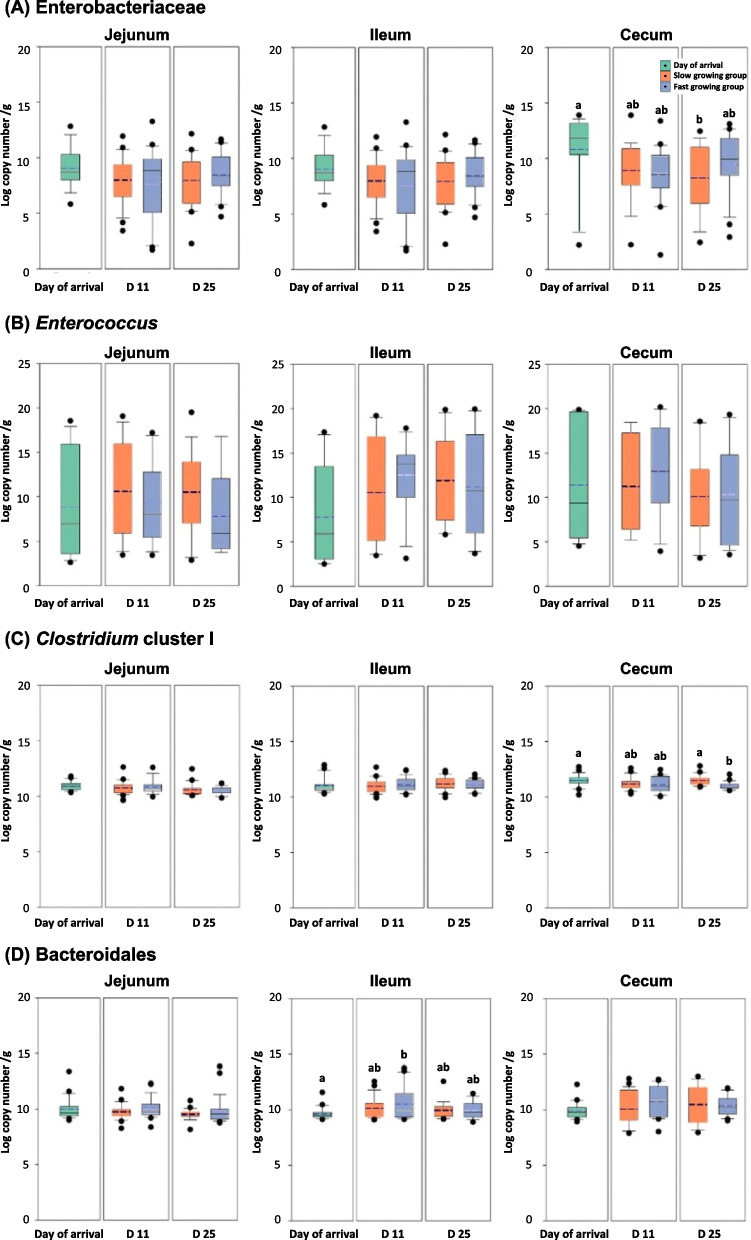
Bacterial populations in mucosa of the intestinal tract (jejunum, ileum, and cecum) analyzed by real-time qPCR. **A** Enterobacteriaceae, (**B**) *Enterococcus*, (**C**) *Clostridium* cluster I, (**D**) *Bacteroidales* at day of arrival, d 11, and d 25. In box plots, square indicates the interquartile range for each data point, and the black and blue lines denote the median and mean values, respectively. The error bars represent the 10^th^ and 90^th^ percentiles and the black circles are outliers. ^a,^^b^ Different letters above the box plots indicate significant differences (*P* < 0.05)

While 16S rRNA amplicon sequencing analysis gave us an opportunity to identify the types of bacteria in the chicken gut, the result is a measurement of diversity not a quantitative analysis. To understand effects of microbial populations on host physiology, we quantified microbial populations using real time qPCR (Fig. 7 and Fig. 8). Data from day of arrival and the slow and fast growing chickens at d 11 and d 25 were compared and statistically analyzed. The total bacterial density of the chicken mucosa did not show significant differences among groups; however, total bacteria in the slow growing chickens tended to be higher than that in the fast growing chickens in the ileum and cecum at d 25 (*P* > 0.05). The trend of lactic acid bacteria populations increased from d 0 to 11 and then decreased. The fast growing chickens showed higher lactic acid bacteria and Bifidobacteria than the slow growing chickens in the ileum at d 25, while there was no significant difference (*P* > 0.05). Likewise, Lactobacillus increased from d 0 to d 11, but there were no clear differences between the slow and fast growing cohorts. Enterobacteriaceae clearly decreased by age, significantly in the cecum (*P* < 0.05). In the case of *Enterococcus*, slow growing chickens tended to have higher populations than fast growing cohorts in the jejunum and ileum at d 25 (*P* > 0.05). While most of the bacterial populations measured by qPCR did not show clear differences among groups because of the large variations in animal samples, a distinct change was observed in *Clostridium* cluster I. In the cecum, slow growing chickens had significantly higher *Clostridium* cluster I at d 25 (*P* < 0.05). *Bacteroidales* increased with age and had higher populations in the fast growing chickens in the ileum at d 11 (*P* < 0.05), while it decreased at d 25.

**Fig. 9:**
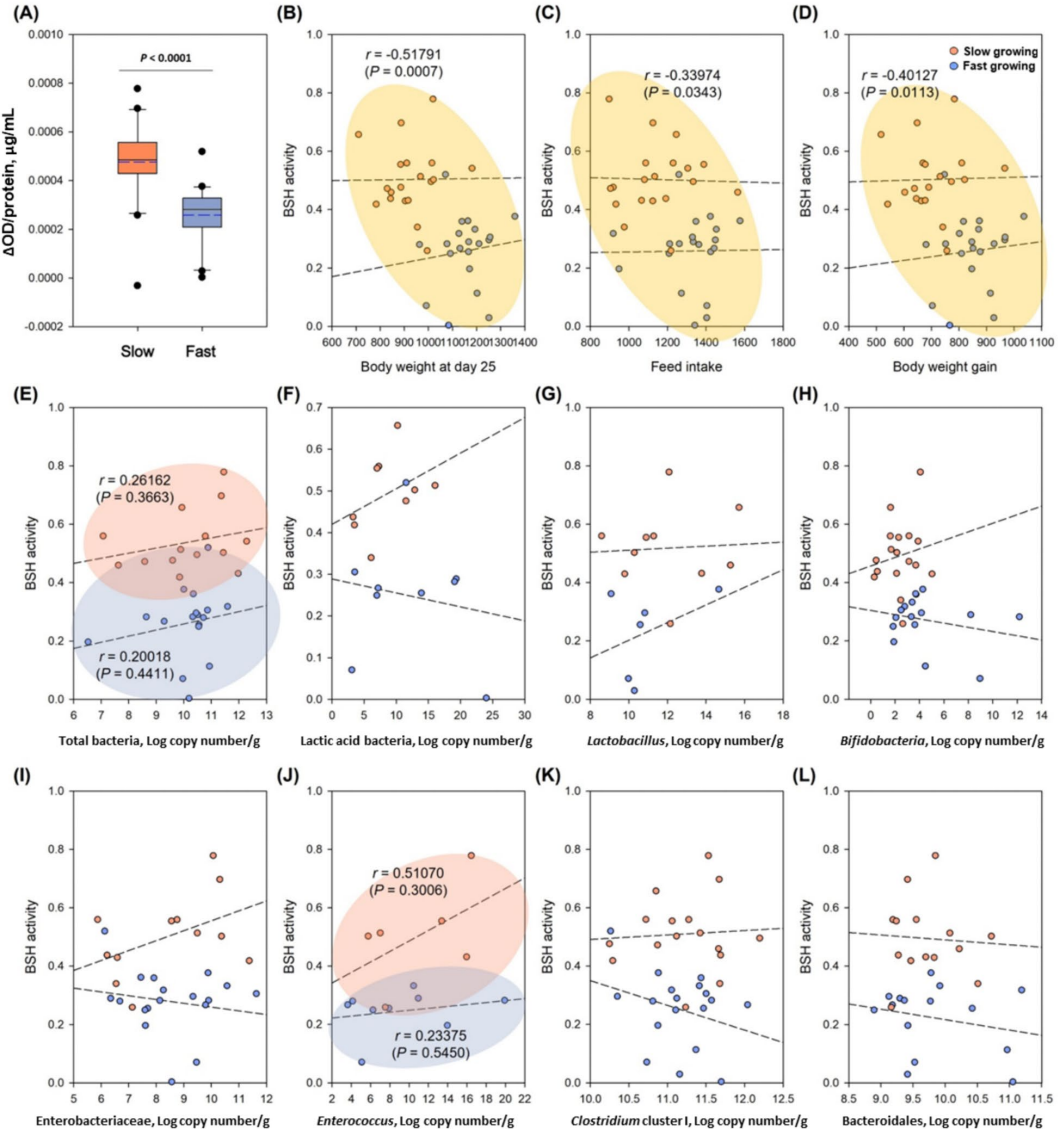
Bile salt hydrolase (BSH) activity and correlation with growth traits and microbiota. **A** BSH activity of ileal samples of chickens in each cohort of slow and fast growing chickens. Values were calculated by the ∆ optical density (OD) between sample and control (negative control, *Clostridium scindens* ATCC 35704; positive control, *Bacteroides vulgatus* ATCC 8482) divided by protein (μg/mL). In box plots, square indicates the interquartile range for each data point, and the black and blue lines denote the median and mean values, respectively. The error bars represent the 10^th^ and 90^th^ percentiles and the black circles are outliers. Values are significantly different (*P* < 0.0001). **B**–**L** Multiple linear regression between the BSH activity and the different variables including (**B**) body weight at d 25, (**C**) feed intake between d 11 and d 25, (**D**) body weight gain between d 11 and d 25, log copy number of (**E**) total bacteria, (**F**) lactic acid bacteria, (**G**) *Lactobacillus*, (**H**) Bifidobacteria, (**I**) Enterobacteriaceae, (**J**) *Enterococcus*, (**K**) *Clostridium* cluster I, and (**L**) Bacteroidales in ileum sample of chickens at d 25

BSH activity was measured from ileum samples of chickens in each slow and fast growing chickens using a synthetic BSH substrate (CA-AMCA) and BCA assay. *Clostridium scindens* and *Bacteroides vulgatus* were used as negative and positive controls, respectively. BSH activity was significantly higher in the slow growing chickens (0.0048 μg/mL) than in the fast growing cohorts (0.0026 μg/mL) (*P* < 0.0001) (Fig. 9).

## Discussion

In commercial poultry production, achieving uniform growth among broiler chickens is challenging despite consistent nutrition and management practices. Our study confirmed that there are significant variations in body weight and feed intake even under controlled conditions, resulting in a distinct growth phenotypes such as fast and slow growing chickens. One of the most consistent patterns we observed was the diverse gut microbial composition and BSH activity between the groups. While overall microbial diversity increased by age, slow growing chickens showed higher levels of specific bacterial populations including *Enterococcus, Clostridium* cluster I, and total bacteria. Another interesting finding was the significant increase in BSH activity in slow growing chickens. In our study, BSH activity was negatively correlated with body weight, feed intake, and body weight gain, supporting the idea that microbial metabolism plays a key role in shaping host growth outcomes.

We observed differences in the relative abundance of Ca. *Arthromitus,* which is poorly characterized and yet uncultured taxon defined by DNA sequence analysis [28]. It is also known as a segmented filamentous bacteria (SFB), which are commensal organisms that attach to the ileal epithelium of host without causing an inflammatory response. Instead, it is known to enhance adaptive and innate immunity of the host through antigen specific Th17 cells and immunoglobulin preventing the colonization of pathogenic microorganisms by blocking epithelial receptors [29]. In a recent microbiome study with turkey, Ca. *Arthromitus* presented in higher proportions in higher-performing flocks, which could possibly enhance host immune response and impact BSH activity resulting in performance outcome changes [30]. It is considered that colonization of SFB in the intestinal tract of poultry is beneficial early in life for immune system development, but its proportion should decrease over time to allow for a mature microbiome [31].

While there was a large variation within the samples, we found some consistent patterns of bacterial populations. For example, levels of lactic acid bacteria measured by qPCR tended to be higher in the slow growing chickens, which is consistent with the bacterial cell count data. Increased number of *Lactobacillus* and higher populations in the small intestine of the slow growing chickens were also consistent with the data (relative abundance in small intestine at d 25: slow, 23.0%; fast 17.9%). In the case of *Bifidobacteria*, it was consistently higher in the ileum of the fast growing chickens in all cohorts (Fig. S5). General trends of microbial population changes revealed that total bacteria, *Enterococcus*, and *Clostridium* cluster I were higher in slow growing chickens and *Bacteroidales* were higher in the ileum of fast growing chickens at d 25.

We postulated that differences in microbial populations between slow and fast growing chickens might be related to microbial metabolism associated with body weight changes in the host. For decades, antibiotic growth promoters (AGP) have been used in poultry feed to improve the growth rate and feed conversion efficiency. The initial concept of AGP was related to their antibacterial mode reducing the total number of gut microbiota [32], resulting in the reduction of microbial metabolites regarding bile acid metabolism [33, 34]. However, due to the concern for the development of antibiotic-resistant bacteria, reduction of the use of in-feed antibiotics in healthy animals has been a worldwide trend. Instead, BSH is regarded as a key mechanistic target for developing alternatives to AGP for enhanced animal production, since the effects of BSH activity is consistent with that of AGP [16].

BSH enzyme produced by intestinal bacteria catalyzes deconjugation of primary bile acids by hydrolyzing the amide bond, which is an essential gateway reaction in the metabolism of bile acids thereby affecting the host fat metabolism [15]. Body weight gain of animals is related to the activity of this enzyme as well as the abundance of potent BSH-producing bacteria [17]. As early as the 1980 s, Feighner and Dashkevicz [35] reported that inhibition of BSH activity could promote feed efficiency and body weight gain in chickens. Guban et al. [36] further confirmed that antibiotic growth promoter treatment improved body weight gain and fat digestibility with reduced BSH activity in the intestine of broiler chickens. A more recent study also concluded that inhibition of BSH activity could enhance body weight gain by altering bile acid pool, host signaling, and lipid metabolism in the chicken model system [37]. BSH activity also plays a role in in vivo adaptation of intestinal microorganisms in the intestinal tract by contributing to the resistance of commensal bacteria towards bile salts [15, 38]. Accordingly, we targeted BSH as a key mechanistic biomarker for uncovering the relationships between gut microbiota and body weight differences in chickens.

BSH activity is known to be distributed widely across the major bacterial divisions in the intestinal tract including *Lactobacillus*, *Bifidobacterium*, *Enterococcus*, *Clostridium* and *Bacteroides* species [39]. BSH genes are particularly abundant in lactic acid bacteria such *Lactobacillus* and *Bifidobacteria*, but high levels of BSH activity in *E. faecium* and *C. perfringens* were also reported [15, 40, 41]. In this study, BSH activity showed significantly negative correlations with body weight (*r* = −0.5179, *P* = 0.0007), feed intake (*r* = −0.33974, *P* = 0.0343), and body weight gain (*r* = −0.40127, *P* = 0.0113) (Fig. 9). Among the targeted microorganisms by qPCR, only total bacteria and *Enterococcus* tended to show positive correlations with BSH activity in each slow and fast growing chickens. The correlation coefficients (*r*) with total bacteria were 0.2616 and 0.2002 in the slow and fast growing cohorts, respectively, while those with *Enterococcus* were 0.5107 and 0.2338. We concluded that high number of bacteria may affect the high BSH activity in the intestine reducing the lipid emulsification, consequently resulting in reduced body weight (slow growing chickens).

## Conclusion

Our study suggests that specific gut microorganisms, including *Enterococcus*, may influence bile salt hydrolase activity, which in turn affects body weight and feed intake. This highlights the role of microbial bile acid metabolism in host physiology, even among chickens fed with same antibiotic-free diets under the same environmental conditions. However, a limitation of this study is the weak and non-significant correlations between BSH activity and target microorganisms identified by qPCR. This suggests that other BSH-containing microorganisms, such as Ca. *Arthromitus*, not quantified in this study, might also contribute to BSH activity and its impact on host physiology. Thus, future studies such as transcriptomic analysis and metagenome-assembled genome analysis targeting the BSH and lipid metabolism can be another approach to elucidate more detail of the relationship between gut microbiota and body weight differences in chickens. The findings from this study suggest potential factors contributing to the variability in broiler chickens, ultimately implicating valuable insights for enhancing production efficiency in the poultry industry.

## Supplementary Information

Additional file 1: Table S1. Composition of the experimental diet.

Additional file 2: Table S2. PCR conditions for library preparation.

Additional file 3: Table S3. Primers used for real time qPCR in this study.

Additional file 4: Table S4. Real-time qPCR standard curves for DNA quantification of the samples.

Additional file 5: Table S5. Gain:feed and feed conversion ratioof fast and slow growing cohorts during the grower phase.

Additional file 6: Fig. S1. Shannon index in each groups according to cohort, days, groups, and site of GI tract. FS1 to 3: cohort number 1 to 3; 11 and 25: d 11 and d 25; f and s: fast and slow growing group; s and c: small intestine and cecum.

Additional file 7: Fig. S2. Concentrations of acetate, propionate, and butyrate inileal andcecal contents of slow and fast growing chickens at d 25.

## Data Availability

The sequences have been deposited at the NCBI under the submission number SUB14821259 (Bioproject ID: PRJNA1187796).

## Abbreviations

AGP: Antibiotic growth promoters
APEC: Avian pathogenic *E. coli*
BSH: Bile salt hydrolase
GMM: Gut microbiota medium
LAB: Lactic acid bacteria
qPCR: Quantitative PCR
