# Supplementary material for: Intestinal microbiota composition and bile salt hydrolase activity in fast and slow growing broiler chickens: implications for growth performance and production efficiency
Source: J Anim Sci Biotechnol. 2025 Aug 2;16:108. doi: 10.1186/s40104-025-01243-4 (PMC12317501; doi:10.1186/s40104-025-01243-4)
Supplement: Supplementary file 1 — Additional file 1: Table S1. Composition of the experimental diet. [file 40104_2025_1243_MOESM1_ESM.docx]

**Table S1** Composition of the experimental diet

| **Ingredient** | **Concentration, g/kg** |
| --- | --- |
| Corn | 525.8 |
| Soybean meal | 390.0 |
| Soy oil | 35.0 |
| Sodium chloride | 4.0 |
| Limestone | 12.0 |
| Dicalcium phosphate | 21.0 |
| Vitamin premix^2^ | 2.0 |
| Mineral premix^3^ | 1.5 |
| Choline chloride | 3.2 |
| L-Lys HCl | 1.4 |
| DL-Met | 3.2 |
| L-Thr | 0.9 |
| Calculated composition |  |
| Protein, g/kg | 229.3 |
| ME, kcal/kg | 3046 |
| Ca, g/kg | 10.6 |
| Total P, g/kg | 8.0 |
| nPP, g/kg | 5.3 |
| Ca:tP | 1.3 |
| Ca:nPP | 2.0 |
| Na, g/kg | 2.0 |
| Total AA, g/kg |  |
| Arg | 14.3 |
| His | 5.8 |
| Ile | 8.8 |
| Leu | 17.3 |
| Lys | 12.7 |
| Met | 6.4 |
| Met + Cys | 9.5 |
| Phe | 10.3 |
| Phe + Tyr | 16.3 |
| Thr | 8.3 |
| Trp | 2.5 |
| Val | 9.5 |
| ^1^Abbreviations: nPP = non-phytate phosphorous; tP = total phosphorous; ME = metabolizable energy  ^2^Provided per kilogram of complete diet: retinyl acetate, 4,400 IU; cholecalciferol, 25 μg; dl-α-tocopheryl acetate, 11 IU; vitamin B12, 0.01 mg; riboflavin, 4.41 mg; d-Ca-pantothenate, 10 mg; niacin, 22 mg; and menadione sodium bisulfite complex, 2.33 mg  ^3^Provided per kilogram of complete diet: Mn, 75 mg from MnO; Fe, 75 mg from FeSO_4_·7H_2_O; Zn, 75 mg from ZnO; Cu, 5 mg from CuSO_4_·5H_2_O; I, 0.75 mg from ethylenediamine dihydroiodide; and Se, 0.1 mg from Na_2_SeO_3_ | |
