# Supplementary material for: Intestinal microbiota composition and bile salt hydrolase activity in fast and slow growing broiler chickens: implications for growth performance and production efficiency
Source: J Anim Sci Biotechnol. 2025 Aug 2;16:108. doi: 10.1186/s40104-025-01243-4 (PMC12317501; doi:10.1186/s40104-025-01243-4)
Supplement: Supplementary file 2 — Additional file 2: Table S2. PCR conditions for library preparation. [file 40104_2025_1243_MOESM2_ESM.docx]

**Table S2** PCR conditions for library preparation

**PCR Stages Number of Cycles**

50ºC 2 minutes 1

70ºC 20 minutes 1

95ºC 10 minutes 1

95ºC 15 seconds

55ºC 30 seconds

72ºC 1 minute 10

95ºC 15 seconds

80ºC 30 seconds

60ºC 30 seconds

72ºC 1 minute 2

95ºC 15 seconds

55ºC 30 seconds

72º 1 minute 8

95ºC 15 seconds

80ºC 30 seconds

60ºC 30 seconds

72ºC 1 minute 2

95ºC 15 seconds

55ºC 30 seconds

72ºC 1 minute 8

95ºC 15 seconds

80ºC 30 seconds

60ºC 30 seconds

72ºC 1 minute 5
