# Supplementary material for: Intestinal microbiota composition and bile salt hydrolase activity in fast and slow growing broiler chickens: implications for growth performance and production efficiency
Source: J Anim Sci Biotechnol. 2025 Aug 2;16:108. doi: 10.1186/s40104-025-01243-4 (PMC12317501; doi:10.1186/s40104-025-01243-4)
Supplement: Supplementary file 3 — Additional file 3: Table S3. Primers used for real time qPCR in this study. [file 40104_2025_1243_MOESM3_ESM.docx]

**Table S3** Primers used for real time qPCR in this study

| **Target** | **Primer sequence** | **Reference** |
| --- | --- | --- |
| Total bacteria | For: GTGYCAGCMGCCGCGGTAA | Walters et al. [1] |
|  | Rev: GGACTACNVGGGTWTCTAAT |  |
| Lactic acid bacteria | For: GGCGGCGTGCCTAATACATGCAAGT | Bokulich and Millis [2] |
|  | Rev: TCGCTTTACGCCCAATAAATCCGGA |  |
| *Lactobacillus* | For: CACCGCTACACATGGAG | Walter et al. [3] |
|  | Rev: AGCAGTAGGGAATCTTCCA |  |
| *Bifidobacterium* spp. | For: CGCGTCYGGTGTGAAAG | Peinado et al. [4] |
|  | Rev: CCCCACATCCAGCATCCA |  |
| *Enterococcus* | For: CCCTTATTGTTAGTTGCCATATT | Rintilla et al. [5] |
|  | Rev: ACTCGTTGTACTTCCCATTGT |  |
| *Clostridium* cluster I | For: TACCHRAGGAGGAAGCCAC | Boroojeni et al. [6] |
|  | Rev: GTTCTTCCTAATCTCTACGCAT |  |
| Bacteroidales | For: GGTGTCGGCTTAAGTGCCAT | Lunedo et al. [7] |
|  | Rev: CGGAYGTAAGGGCCGTGC |  |

**References**

1. Walters W, Hyde ER, Berg-Lyons D, Ackermann G, Humphrey G, Parada A, et al. Improved bacterial 16S rRNA gene (V4 and V4-5) and fungal internal transcribed spacer marker gene primers for microbial community surveys. mSytems. 2016;1(1):e00009-15.
2. Bokulich NA, Mills DA. Differentiation of mixed lactic acid bacteria communities in beverage fermentations using targeted terminal restriction fragment length polymorphism. Food Microbiol. 2012;31(1):126-132.
3. Walter J, Tannock GW, Tilsala-Timisjarvi A, Rodtong S, Loach DM, Munro K, et al. Detection of *Lactobacillus, Pediococcus, Leuconostoc*, and *Weissella* species in human feces by using group-specific PCR primers and denaturing gradient gel electrophoresis. Appl Environ Microbiol. 2001;67(6):2578-2585.
4. Peinado MJ, Ruiz R, Echávarri A, Aranda-Olmedo I, Rubio LA. Garlic derivative PTS-O modulates intestinal microbiota composition and improves digestibility in growing broiler chickens. Anim Feed Sci Technol. 2013;181:87–92.
5. Rinttilä T, Kassinen A, Malinen E, Krogius L, Palva A. Development of an extensive set of 16S rDNA-targeted primers for quantification of pathogenic and indigenous bacteria in faecal samples by real-time PCR. J Appl Microbiol. 2004;97(6):1166-1177.
6. Boroojeni FG, Vahjen W, Männer K, Knorr F, Ruhnke I, Röhe I, et al. The effects of different thermal treatments and organic acid levels on nutrient digestibility and microbial composition in broilers fed wheat-based diets. Poult Sci 2014;93(7):1848-1857.
7. Lunedo R, Furlan LR, Fernandez-Alarcon MF, Squassoni GH, Campos DMB, Perondi D, et al. Intestinal microbiota of broilers submitted to feeding restriction and its relationship to hepatic metabolism and fat mass: Fast-growing strain. J Anim Physiol Anim Nutr (Berl). 2019;103(4):1147-1157.
