# Supplementary material for: Intestinal microbiota composition and bile salt hydrolase activity in fast and slow growing broiler chickens: implications for growth performance and production efficiency
Source: J Anim Sci Biotechnol. 2025 Aug 2;16:108. doi: 10.1186/s40104-025-01243-4 (PMC12317501; doi:10.1186/s40104-025-01243-4)
Supplement: Supplementary file 4 — Additional file 4: Table S4. Real-time qPCR standard curves for DNA quantification of the samples. [file 40104_2025_1243_MOESM4_ESM.docx]

**Table S4** Real-time qPCR standard curves for DNA quantification of the samples

| **Target organisms** | **Equation** | **R-square** |
| --- | --- | --- |
| Total bacteria | y = -3.8659x + 52.443 | R² = 0.9874 |
| Lactic acid bacteria | y = -1.4819x + 39.68 | R² = 0.9904 |
| *Lactobacillus* | y = -4.0196x + 63.292 | R² = 0.9404 |
| *Bifidobacterium* spp. | y = -1.7552x + 32.258 | R² = 0.9109 |
| Enterobacteriaceae | y = -1.9481x + 36.78 | R² = 0.9519 |
| *Enterococcus* | y = -1.9118x + 40.741 | R² = 0.9393 |
| *Clostridium* cluster I | y = -10.093x + 118.68 | R² = 0.9548 |
| Bacteroidales | y = -5.4566x + 70.546 | R² = 0.9226 |
