# Supplementary material for: Intestinal microbiota composition and bile salt hydrolase activity in fast and slow growing broiler chickens: implications for growth performance and production efficiency
Source: J Anim Sci Biotechnol. 2025 Aug 2;16:108. doi: 10.1186/s40104-025-01243-4 (PMC12317501; doi:10.1186/s40104-025-01243-4)
Supplement: Supplementary file 5 — Additional file 5: Table S5. Gain:feed and feed conversion ratioof fast and slow growing cohorts during the grower phase. [file 40104_2025_1243_MOESM5_ESM.docx]

**Table S5.** Gain:feed (g/kg) and feed conversion ratio (FCR, g/g) of fast and slow growing cohorts during the grower phase (d 11-25)

|  | | **Gain:feed, g/kg** | **FCR, g/g** |
| --- | --- | --- | --- |
| Mean | Slow growing group | 631 (n = 29) | 1.60 (n = 28) |
|  | Fast growing group | 671 (n = 30) | 1.52 (n = 30) |
| Standard error of the mean | | 18.8 | 0.05 |
| *P*-value | | 0.14 | 0.19 |
