# Supplementary material for: Intestinal microbiota composition and bile salt hydrolase activity in fast and slow growing broiler chickens: implications for growth performance and production efficiency
Source: J Anim Sci Biotechnol. 2025 Aug 2;16:108. doi: 10.1186/s40104-025-01243-4 (PMC12317501; doi:10.1186/s40104-025-01243-4)
Supplement: Supplementary file 6 — Additional file 6: Fig. S1. Shannon index in each groups according to cohort, days, groups, and site of GI tract. FS1 to 3: cohort number 1 to 3; 11 and 25: d 11 and d 25; f and s: fast and slow growing group; s and c: small intestine and cecum. [file 40104_2025_1243_MOESM6_ESM.docx]

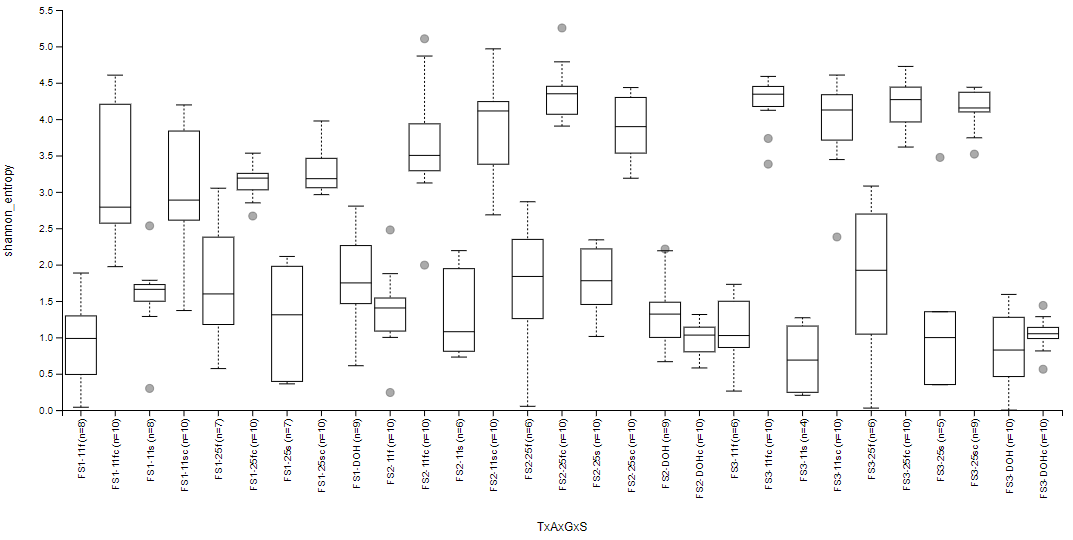


**Fig. S1.** Shannon index in each groups according to cohort, days, groups, and site of GI tract. FS1 to 3: cohort number 1 to 3; 11 and 25: d 11 and d 25; f and s: fast and slow group; s and c: small intestine and cecum.
