# Supplementary material for: Intestinal microbiota composition and bile salt hydrolase activity in fast and slow growing broiler chickens: implications for growth performance and production efficiency
Source: J Anim Sci Biotechnol. 2025 Aug 2;16:108. doi: 10.1186/s40104-025-01243-4 (PMC12317501; doi:10.1186/s40104-025-01243-4)
Supplement: Supplementary file 7 — Additional file 7: Fig. S2. Concentrations of acetate, propionate, and butyrate inileal andcecal contents of slow and fast growing chickens at d 25. [file 40104_2025_1243_MOESM7_ESM.docx]

**
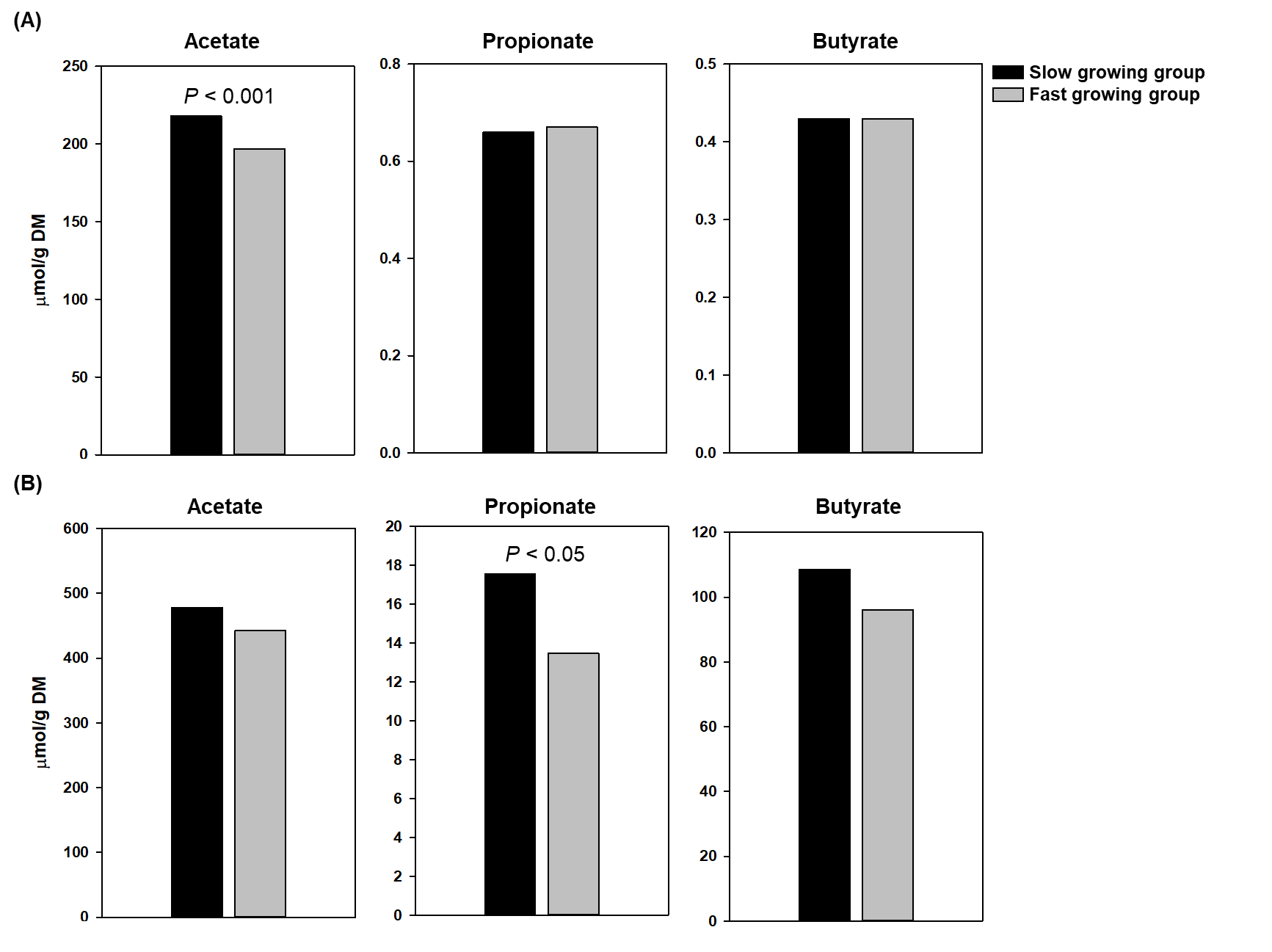
**

**Fig. S2.** Concentrations of acetate, propionate, and butyrate in (A) ileal and (B) cecal contents of slow and fast growing chickens at d 25.
